# Clarifying the Normative Significance of ‘Personality Changes’ Following Deep Brain Stimulation

**DOI:** 10.1007/s11948-020-00207-3

**Published:** 2020-03-18

**Authors:** Jonathan Pugh

**Affiliations:** Oxford Uehiro Centre for Practical Ethics, University of Oxford, Suite 8, Littlegate House, St Ebbes Street, Oxford, OX1 1PT UK

**Keywords:** Deep Brain Stimulation, Personality, Self, Identity, Authenticity, Autonomy, Agency

## Abstract

There is evidence to suggest that some patients who undergo Deep Brain Stimulation can experience changes to dispositional, emotional and behavioural states that play a central role in conceptions of personality, identity, autonomy, authenticity, agency and/or self (PIAAAS). For example, some patients undergoing DBS for Parkinson’s Disease have developed hypersexuality, and some have reported increased apathy. Moreover, experimental psychiatric applications of DBS may intentionally seek to elicit changes to the patient’s dispositional, emotional and behavioural states, in so far as dysfunctions in these states may undergird the targeted disorder. Such changes following DBS have been of considerable interest to ethicists, but there is a considerable degree of conflict amongst different parties to this debate about whether DBS really does change PIAAAS, and whether this matters. This paper explores these conflicting views and suggests that we may be able to mediate this conflict by attending more closely to what parties to the debate mean when they invoke the concepts lumped together under the acronym PIAAAS. Drawing on empirical work on patient attitudes, this paper outlines how these different understandings of the concepts incorporated into PIAAAS have been understood in this debate, and how they may relate to other fundamental concepts in medical ethics such as well-being and autonomy. The paper clarifies some key areas of disagreement in this context, and develops proposals for how ethicists might fruitfully contribute to future empirical assessments of apparent changes to PIAAAS following DBS treatment.

The early 2000s saw the publication of three papers detailing the results of interview studies involving patients undergoing Deep Brain Stimulation (DBS) for the treatment of movement disorders associated with Parkinson’s Disease (Agid et al. 2006; Houeto et al. 2002; Schüpbach et al. 2006). These papers have had a significant impact on ethical debate in this area due to their reports of participants experiencing personal, social and behavioural difficulties adjusting to DBS treatment (Agid et al. 2006; Schüpbach et al. 2006). In particular, these papers suggested that some patients experienced a sense of self-estrangement following treatment, with some patients reporting that they ‘no longer recognise themselves’ (Agid et al. 2006) or that ‘I do not feel like myself anymore’ following DBS treatment (Schüpbach et al. 2006). As a result, there have been a plethora of articles in the neuroethics literature since, addressing the ethical implications of this phenomenon, and an increasing number of patient interview studies investigating it.

This is perhaps unsurprising. The brain has a crucial role in “the functioning of the mind, the body, and the development of self conceptions and autonomous agency” (Nuffield Council of Bioethics 2013, p. 4.12), and people are thus likely to be particularly concerned about deleterious effects of direct brain interventions on these highly valued aspects of human life. However, as Frederic Gilbert and colleagues have recently argued, the ethical discussion of these putative effects of DBS has arguably outstripped clinical reality. They claim that the debate relies on limited empirical evidence and is often based on unsubstantiated speculative assumptions, both about the extent of this problem, and about the causal role that DBS itself is playing (Gilbert et al. 2018). More recently, (Bluhm et al. 2019) have argued that much of the neuroethical discussion wrongly assumes that the concerns raised by extreme cases of apparent personality change following DBS (involving the onset of mania for example) only differ in degree from the concerns about personality change that typical DBS patients face. These authors instead argue that these extreme cases in fact raise differ *kinds* of concerns to the one’s patients typically face when adjusting to DBS treatment.

Whilst these are provocative claims, this paper shall not challenge them. First, even if one agrees that there is limited empirical evidence of changes to PIAAAS, they may still be understood as normatively significant; indeed, Gilbert and colleagues themselves claim that these effects remain a critical ethical concern despite their claims about the empirical grounds of the neuroethical debate (Gilbert et al. 2018).^1^ Second, a plausible explanation for why the neuroethical literature might make the assumption that Bluhm and colleagues attack, is that ethicists fail to be sufficiently fine-grained about the moral concepts that they bring to bear on these different cases. Indeed, Bluhm and colleagues themselves explicitly leave open the question of “whether changes to an individual’s authenticity, agency, and the like, should be understood as changes to aspects of personal identity or as affecting distinct, though related, aspects of the self”, and instead use the term “self-related characteristics” to encompass all of them. (Bluhm et al. 2019, footnote 2). The problem with this strategy is that if we lump these disparate concepts under such broad umbrella terms, it can be difficult to draw neat distinctions between the different kinds of cases and concerns that Bluhm et al. highlight.

Similar conflations are apparent in the wider ethical literature. In their review, (Gilbert et al. 2018) searched for papers that discuss changes to personality, identity, autonomy, agency, authenticity, and/or self under the acronym PIAAAS. As they note, all of these concepts have been invoked in the debate, often in the discussion of the same series of case reports. The empirical literature also lumps together these concepts. In a recent patient interview study, Sanneke de Hann and colleagues acknowledge that there may be technical differences between the concepts of personality, self and personal identity, but employ these terms interchangeably in their study (de Haan et al. 2017).

I do not doubt the utility of bringing these concepts under one umbrella for the purpose of analyzing broad trends in the literature, or for interpreting the remarks of patients whose comments are unlikely to be grounded by a deep philosophical understanding of the distinctions between these concepts. Moreover, it is plausible to suggest that these somewhat overlapping concepts are likely to be relevant to the same sorts of practical moral concerns. Nonetheless, these are distinct, albeit related ethical concepts, and lumping them together in theoretical discussions of this phenomenon can serve to distort the ethical debate that they undergird.

There is a further important question that has been identified in this area that this paper shall also not address, namely the causal question regarding whether DBS is alone responsible for directly inducing the changes observed in these patients (Pugh et al. 2018; Bluhm et al. 2019). Changes to PIAAAS amongst these patients could be explained by a number of other contributing factors, including (amongst others) underlying disease progression or the so-called burden of normality (Gilbert 2012). A complete ethical analysis of these changes should engage with this causal question; the reason for this is that it may be that we should be more concerned with changes that are directly induced by DBS than those that are not a direct effect of stimulation (Baylis 2013; Bluhm et al. 2019).

Whilst acknowledging this important point, this paper shall set this issue aside for two reasons. First, the answer to the causal question must be determined empirically, and there are a number of significant challenges to empirically establishing a direct causal relationship between DBS treatment and changes to PIAAAS (Pugh et al. 2018). Second, changes to PIAAAS can have moral significance even if their causal history is uncertain. The motivation for this paper is that it is important to be clear about the nature and normative significance of the phenomenon *itself*, before getting to the deeper questions about causality and the further implications that this might have. Changes to PIAAAS might matter morally per se, even if it is true they are *more* problematic when they are directly induced by neurostimulation.

Instead, this paper shall address a problem with the way in which ethical discussions about the normative implications of these effects has so far been parsed. More specifically, it will address the considerable divergence of philosophical concepts that ethicists have used in their analysis of the available empirical evidence. This in itself is not problematic; the use of different concepts, and indeed different understandings of the same concept can often help to illuminate different aspects of the same phenomenon. However, as this paper shall explain, the ethical literature on DBS evidences deep disagreements about the nature of key concepts, and theorists do not always elucidate their understanding of relationships between different concepts, and the normative implications that this can have. In particular, it is often unclear precisely how these concepts should be understood to relate to moral principles that are typically employed in framing debates in medical ethics, such as beneficence, non-maleficence and respect for autonomy. It is an open question as to whether changes to PIAAAS will require us to alter or understanding of the nature and role of these principles, or whether they are already equipped to satisfactorily address the moral questions in this area.

However, this is not just a theoretical concern. It is becoming clearer that there is a need for further empirical data about changes to PIAAAS following DBS (Gilbert et al. 2018). Since the concepts included under this acronym are ethical, rather than clinical concepts, ethicists can and should have a central role to play in helping to develop the sort of investigative tools that will be necessary to fully illuminating this phenomenon, and to bridging the gap between the empirical and normative debates in this area. Yet, if ethicists are to do so, then they must strive for greater clarity with regards to these concepts, and, just as importantly, their normative significance. Failure to do so is to risk becoming engrossed in esoteric theoretical squabbles grounded by the use of different ill-defined terms for the same phenomena. This is a surefire way to confuse and alienate clinicians who are keenly aware of the potential ethical issues in this area.

My aim in this paper is to take some tentative steps in this regard, by mapping out the conceptual theoretical terrain in this context, with a view to supporting some recommendations for how philosophical ethics may fruitfully inform empirical investigations into changes to PIAAAS following DBS. To be clear, the aim of this paper is not to defend or endorse a particular theory of an element of PIAAAS, although this paper shall offer some critical comments on this score. Rather, by bringing together various strands of the literature on this topic, this paper aims to (1) elucidate the different ways in which DBS may have normatively significant effects, depending on the understanding of the above concepts employed, and (2) to ground some practical recommendations on the basis of this analysis.

The discussion shall begin with a distinction between two different forms of identity, which has been widely (though not universally) acknowledged in the existing literature. It shall then go on to consider more complex distinctions and relations between the other concepts that fall within the category of PIAAAS.

## Not All Identities are Equal: Numerical and Narrative Identity

A considerable number of theorists invoke the concept of identity in their discussions of psycho-socical changes following DBS (Baylis 2013; Goddard 2017; Lipsman and Glannon 2013; Müller et al. 2017; Witt et al. 2013). This is quite natural, given that some patients have used the phrase of “becoming a different person” following DBS treatment (de Haan et al. 2017). However, it is widely agreed that there are (at least) two different senses in which it is possible to understand identity from a philosophical perspective: first, identity in the ‘numerical’ sense, and second, identity in the ‘narrative’ sense. As this section of the paper shall explain, the latter sense of identity is far more pertinent to the DBS debate than the former. To their credit, a number of theorists have explicitly recognised this distinction in their discussions, and explained the sense of identity that they mean to invoke.^2^ However, for reasons that will become apparent, a recent critique of the ethical debate on identity suggests that this distinction bears repeating.

Numerical identity is the sense of identity that is invoked in discussions about what it is for something to exist through time as the same thing; the concept of personal identity in this sense aims to provide an answer to what Marya Schechtmann calls the “re-identification question”, which is “what makes a person at time t1 the same person as a person at t2?” (Schechtman 1996) This concept of identity has been a central concern throughout the history of philosophy. For the purposes of this paper though, it will suffice to draw a basic distinction between substance-based views, and psychological views of numerical identity.

The former type of theory holds that numerical identity is a matter of existing as the same substance. Whilst such a view might appeal to a non-material soul to ground claims about identity, contemporary substance-based views commonly claim that the crucial substance for numerical identity is the *biological* substance. For instance, one particularly prominent approach, termed ‘animalism’, claims that human persons are “… essentially animals, living members of the species Homo Sapiens” (DeGrazia 2005, p. 48). On this account, what makes a person at time t1 the same person as a person at t2 is simply that they exist as the same human animal (DeGrazia 2005; Olson 1999).

In contrast, psychological theories deny that numerical identity requires the persistence of any particular substance, and instead claim that this sense of identity depends on the psychological relationship between a person at one time and another. Whilst these theories can be traced back to John Locke, Derek Parfit’s psychological account has been the most widely discussed in the DBS literature. According to Parfit, psychological continuity is a necessary (though not sufficient)^3^ condition of personal identity, where psychological continuity involves a person having overlapping chains of strong psychological connectedness across time (Parfit 1984, p. 205). This requires some unpacking. First, ‘psychological connectedness’ requires holding direct psychological connections across time; these direct psychological connections can include those that hold between memory of an event and its actual occurrence, an intention and its moving one to act, or merely holding a belief or desire over time. A person achieves *strong* psychological connectedness (of the sort that psychological continuity requires) if “the number of direct connections over any day, is at least half the number that hold over every day, in the lives of nearly every actual person” (Parfit 1984, p. 206).

Numerical identity, or at least the psychological continuity that is a necessary condition of it on the Parfitian approach,^4^ is foundational to many of the things that matter morally. First, it undergirds an individual’s prospective self-interest; whilst individuals may care a great deal about the well-being of other people, they typically have a particular concern for their *own* future well-being. Yet for such prospective self-interest to make sense, one must presume that one is numerically identical with some future person who is the object of one’s concern. To illustrate the point, if Smith lacked numerical identity with any future person at time t2, it is not simply the case that this would negatively affects Smith’s well-being because the process of losing his identity would be an unpleasant experience (although it might be); rather, it would mean that at t2 Smith would have ceased to exist as a subject of well-being *at all*. For similar reasons, valid ascriptions of retrospective responsibility for an action in the past must also assume numerical identity; just as it would plausibly be wrong to punish an innocent person for a crime committed by a guilty person, so too would it be wrong to punish a person who is not the same as the person who committed a crime.

A recent paper decrying the encroachment of theories of identity on neuroethical debates (including DBS) highlights the need to be clear about these normative implications (Müller et al. 2017). In their discussion, Sabine Müller and colleagues rightly point out that current medico-legal practice is primarily grounded by something like an animalist theory of identity. They worry that controversial competing psychological theories of identity would require thorough revisions of legal instruments, such as advance directives and so-called Ulysees contracts in DBS treatment.

Nonetheless, they also claim that the law is “metaphysically neutral”, in so far as it respects the metaphysical stance of both animalists and psychological theorists (Müller et al. 2017, p. 100). Their explanation for this claim is that if one endorses an animalist theory, then any treatment wishes enshrined in an advance directive will be respected even if one loses psychological continuity with one’s earlier self. Further, those who endorse psychological theories can simply choose to not to write an advance directive (Müller et al. 2017).

This point about the metaphysical neutrality of the law is well-taken; however it is supplemented with the following more problematic claims.

If revisionary metaphysical theory and common medico-legal practice are in conflict, practice wins over theory if the revisionary metaphysics is based on controversial assumptions and if the practice is beneficial to patients. Advance directives are beneficial to patients because they are effective legal instruments that allow patients to exercise their autonomy (Müller et al. 2017, p. 300).

These further claims concerning the implications of particular medico-legal practices for beneficence are problematic because they flagrantly beg the question against psychological theories of numerical identity. One of the main normative implications of these theories is that the kinds of medico-legal practices that the authors refer to here *cannot* be beneficial to patients who are not psychologically continuous with the person who created the directive. On a psychological approach, the person who made the contract or signed the directive has ceased to exist as a subject of well-being; and the directive or contract may be harmful to the new subject of well-being that now exists, for instance, if it instructs the with-holding of life-saving treatment.

It is true that psychological theories of identity are metaphysically controversial, and that the law should seek metaphysical neutrality as far as is possible. However, to claim that these practices are beneficial (*contra* psychological theories) as part of the justification for abandoning revisionary metaphysics, is simply to assume that psychological theories are incorrect at the outset. Whatever the merits of this claim, it is not, and cannot be metaphysical neutral.

Ruptures to numerical identity have highly significant ethical implications. However, they also do not come about easily on either of the approaches considered here. Consider first Parfit’s psychological account. In particular, recall that on this theory, an individual must retain a sufficient number of direct psychological connections across time to maintain their personal identity, where sufficiency is defined with reference to the comparison class of other actual persons. A rupture will only occur on this account if one has less than half the number of connections that hold over every day, in the lives of nearly every actual person. This is a high threshold: whilst ruptures to numerical identity may be evinced by severe neurodegenerative diseases like Alzheimer’s, persons can also lose a considerable number of psychological connections whilst retaining numerical identity with a future person. Furthermore, on biological substance accounts, even the severe losses of strong psychological connectedness associated with Alzheimer’s will not be sufficient. One will only fail to retain numerical identity with a future person when one ceases to be a living human animal.

The upshot of this is that it is highly unlikely that DBS would threaten numerical identity. Even if it is assumed that DBS can change certain psychological features, this may simply be irrelevant to substance-based theories. Furthermore, although some commentators have suggested scenarios in which it might be plausible to suggest that DBS would threaten a patient’s numerical identity on psychological theories (Klaming and Haselager 2010, p. 534), there is little evidence to suggest that DBS would typically have global effects on patients’ psychological economies of the sort that would threaten a sufficient number of psychological connections for this to be the case.^5^

For this reason, it is far from clear that such theories are the central concern in discussions of the effect of DBS on identity, in all but the very most extreme cases. In their disparaging remarks about the influence of metaphysical theories of identity on neuroethical debates, Müller and colleagues suggest that the invocation of these theories is widespread, citing a number of examples (Müller et al. 2017, pp. 302–308). However, contrary to their analysis, many of the examples of cases in which they claim theorists have invoked psychological theories of metaphysical identity are more plausibly understood as cases in which theorists are invoking theories of *narrative*, rather than numerical identity (Nyholm 2018).

Whilst numerical identity is concerned with *what* persists over time, narrative identity pertains to identity in a broader sense, concerning *who* a person is. As Nyholm (2018) points out, it is an ethical rather than metaphysical conception of identity, one that seeks to answer what Schechtmann calls the “characterization question”: “which beliefs, values, desires, and other psychological features make someone the person she is?” (Schechtman 1996, p. 2).

How should the characterisation question be answered? According to Schechtmann herself, the answer is to be found in the narratives that people tell about themselves to make sense of who they are. On this approach, identities are inherently dynamic; individuals constantly change and evolve, but they make sense of themselves by bringing together these changes into a coherent self-narrative. However, there are two important constraints on the kinds of narrative that can constitute identity in this sense on Schechtman’s view. First, according to the ‘articulation constraint’, an individual must be capable of articulating the explanation for why they have adopted the narrative that they have adopted (Schechtman 1996, p. 120). Second, according to the ‘reality constraint’, the identity-constituting narrative must fundamentally cohere with facts about human beings and their environments (Schechtman 1996, p. 120). Francois Baylis has also adopted a narrative approach to inform her relational account of identity, according to which the narratives that constitute identity are developed in and shaped by personal relationships, and embodied experience. Baylis’ approach thus explicitly extends the narrative approach beyond the personal sphere and into the interpersonal (Baylis 2013).

If DBS threatens identity at all, it is far more plausible that it might threaten narrative identity than numerical identity. Schechtman (2010) herself argues that DBS can threaten narrative identity because changes evinced by DBS may not admit of local articulation; the problem here is that if changes to one’s motivations are a direct result of DBS rather than natural personal development, then it is unclear that the individual will be able to adequately explain these changes. Furthermore, the individual could not construe these changes as part of the natural development of their narrative whilst meeting the demands of the reality constraint. Notice that the answer to the causal question alluded to in the introduction to this paper becomes particularly relevant here; if DBS directly induces changes to PIAAAS, then it is perhaps difficult to see how these changes can be incorporated into a self-narrative in a way that satisfies Schechtman’s articulation constraint.

Yet, Baylis is sceptical of the threat that DBS poses to narrative identity in this regard; contra Schechtman, she claims that the patient’s consent to DBS treatment can serve as a form of explanation that would meet the demands of the articulation constraint. Moreover, Baylis worries that if one claims that DBS is a threat to identity, then consistency demands that any and all life events that disrupt the dialectical process of identity-formation, including disease, should be construed as a threat to identity. This would render the claim that DBS threatens identity as “trivially true” (Baylis 2013, p. 523).

**Fig. 1:**
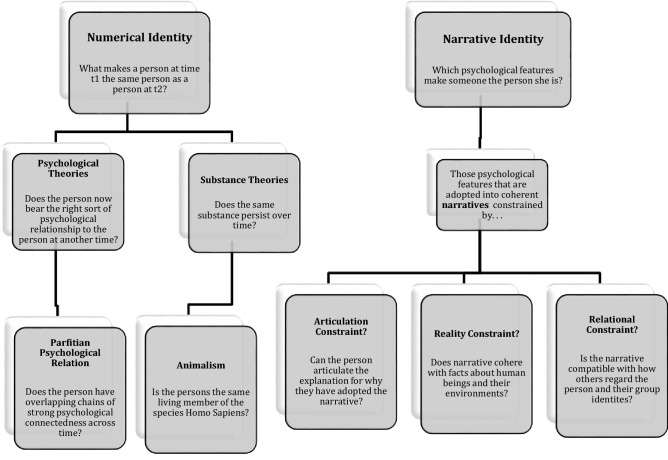
Schematic diagram of the distinction between numerical and narrative identity

This paper shall explore this charge further in the following section. To conclude this discussion concerning the distinction between numerical and narrative identity though, the salient differences between the concepts and the theories surveyed so far can be schematized as follows (Fig. 1).

A few final remarks are apposite. First, whilst there is an important distinction to be drawn between these two senses of identity, there are also important relationships between the two. For one, narrative identity presumes numerical identity for the straightforward reason that one cannot create a self-narrative across time unless one actually exists across that time (DeGrazia 2005, p. 114). Furthermore, many of the psychological features that constitute a person’s self-narrative will be just the same sort of direct psychological connections that are necessary for numerical identity on psychological theories. The difference here is that the loss of a small number of these central psychological features may threaten the coherence of a self-narrative without necessarily threatening strong psychological continuity, which is based on large number of direct psychological connections.

Second, whilst both senses of identity are of normative significance, the reasons for their normative significance differ considerably. A threat to one’s numerical identity is a threat to one’s continued existence as a subject of well-being. Challenges to narrative identity do not threaten one’s status as an extant subject in this way; indeed, it is coherent to say that the loss of core psychological features that partly constitute an agent’s narrative identity is harmful to *that* agent.

This raises the more complex question concerning the nature of narrative identity’s normative significance. This should be answered by considering narrative identity in conjunction with the closely related concepts of personality, self, and authenticity. The reason for this is that although the distinction between narrative and numerical identity is widely endorsed, the neuroethics literature evidences deep and wide-ranging disagreements about the extent to which narrative identity, personality, self and authenticity refer to distinct concepts, with different normative significance.

## The Characterisation Question, Authenticity, Self, and Personality

One might appeal to concepts other than self-narratives in answering the characterization question. For instance, some might be inclined to say that the cluster of psychological features that make a person ‘who they are’ are those that constitute their *personality* or *self*. However, these terms are somewhat slippery, and have been used somewhat loosely in the DBS debate.

There is a considerable degree of overlap between lay understandings of personality and the concept of narrative identity. However, the two are not always understood to be entirely co-extensive. For instance, in discussing the effects of DBS on personality and identity, Walter Glannon suggests that the characteristics that significantly contribute to one’s narrative identity “include the set of dispositional traits we refer to as personality” (Glannon 2009, p. 291). For Glannon then, personality is a narrower concept than narrative identity, which incorporates a wider range of psychological characteristics, and also one’s understanding of one’s own prior experiences.

In stark contrast, Matthis Synofzik and Thomas Schlaepfer claim that personality denotes “a supramodal representational system with largely heterogeneous functional and self-representational levels”, incorporating motor, sensory, and vegetative processes, as well as mental phenomena (Synofzik and Schlaepfer 2008). On this understanding, personality is a far broader concept than narrative identity. Similarly, philosophers have recently brought broad models of the self informed by cognitive science to bear on the DBS debate. For example, Sean Gallagher’s pattern theory of the self incorporates narrative capacities, but also behavioural, affective, embodied and extended elements amongst many others (Dings and de Bruin 2016; Gallagher forthcoming).

Of course, the study of personality is also a significant branch of psychological research, and a number of quite disparate theories of the concept have been generated in this context, rendering it something of a psychological term of art. One of the most influential approaches to the study of personality is “trait theory” which seeks to identify consistent patterns in the way that individuals distinctively think, feel and behave (Cervone 2017, p. 201). However, even this broad approach admits of divergent interpretations. For instance, one can draw a distinction between idiographic theories, which suggest that characteristics are more or less unique to individuals (Allport 1938), and nomothetic theories, which suggest that people merely differ with regards to their position on a continuum of shared broad characteristics (Cattell 1965; Eysenck 1967), such as the so-called “Big Five” personality traits (Cervone 2017, p. Chapter 8). Furthermore, one might distinguish biological theories that suggest that personality is largely a result of inherited dispositions and physiological processes (Eysenck 1967), from others that emphasize environmental influences Bandura (1977).

Despite these different permutations, the trait approach has been operationalized into a number of empirically established personality instruments. However, many of these instruments are not sufficiently fine-grained to detect the sorts of changes that patients report following DBS (Witt et al. 2013). Indeed, the authors of a recent study using several personality scales and semi-structured interviews to assess personality changes following DBS treatment in 27 patients with Parkinson’s Disease found that important changes described in the interviews were not detected by existing quantitative scales (Lewis et al. 2015). So, there is little evidence that DBS changes personality in the sense of the concept that psychologists may employ in developing these tools.

One benefit of bringing these scientifically grounded models of self and personality to bear on the DBS debate is that they are able to offer a more comprehensive understanding of the potential effects that DBS might have. However, this comes at a cost; on such broad models, it is unclear why a change to personality or self has particular normative significance, especially if the model fails to provide an account of a hierarchy between the different elements highlighted by the theory (de Haan et al. 2017). Indeed, some of those who adopt such theories have suggested that the normative questions raised by changes following DBS can be satisfactorily addressed by simply invoking “widely accepted bioethical criteria of beneficence, non-maleficence, and autonomy” (Synofzik and Schlaepfer 2008).

This criticism reflects a deeper theoretical tension regarding the debate surrounding DBS. One the one hand, it may be that an account of the self and/or personality needs to be broad enough to be sensitive to *all* of the potential changes that might occur following DBS treatment. This is particularly important if the primary aim of this discussion is to ensure that patients are sufficiently informed about the potential effects of the procedure, and to capture the concerns that the average patient is likely to face in adjusting to DBS treatment, as highlighted by Bluhm et al. (2019). Yet such concerns are an issue for almost all medical treatments. As such, these broad accounts are vulnerable to the challenge laid down by Baylis against theories of narrative identity: what makes a change to one’s psychological characteristics normatively significant, and why suppose that DBS treatment leads to such changes in particular? There is certainly scope for doubting that the same accounts of the self, personality and narrative identity that are useful for making sense of the concerns that patients typically have adjusting to DBS treatment will be able to play the same role for more extreme cases that arguably raise deeper questions about moral concepts in medicine.

Accordingly, an account of the self and personality might also need to be able to specifically identify those changes that are of particular normative significance, and that perhaps do not admit of straightforward applications of the traditional bioethical criteria of beneficence, non-maleficence and autonomy. There is a legitimate ethical question about which of these goals ought to be prioritised in ethical discussions of DBS. However, the point here is that if one is aiming to develop a suitably sensitive concept that can help us answer the characterization question without being vulnerable to Baylis’ triviality charge, at the very least one must appeal to theoretical apparatus that allows us to distinguish the changes that matter from those that do not.

As explained above, Schechtman attempts to provide such apparatus in the form of her articulation and reality constraints; we should be concerned about changes that are not incorporated into the person’s self-narrative, and/or those elements that fail to meet these constraints. Alternatively, Witt and colleagues propose that changes to a person’s core attitudes are particularly concerning, where a person’s core attitudes are understood to be those attitudes that serve as the foundational function of the agent’s other attitudes (Witt et al. 2013). This is broadly congruous with trait theories of personality, particularly Allsport’s nomothetic account, according to which it is possible to identify central and cardinal traits that are particularly foundational to an individual’s personality (Allport 1938). Yet, there is a crucial difference between Witt and Schechtman’s attempts to determine whether a change is normatively significant; Schechtman focuses on the *process* of the change, whilst Witt and colleagues focus on *what* has been changed.

This difference between process-based and content-based approaches to understanding the normative significance of changes to psychological features following DBS is also reflected in accounts of authenticity. Authenticity is also incorporated into PIAAAS, and has also been understood to be co-extensive with personality in some parts of the literature on DBS. Theories of authenticity are concerned with identifying the ‘true self’ that lies amidst more or less peripheral elements of the self (captured by broader theories, such as Gallagher’s pattern theory) and the conditions under which that self can undergo change, if at all.^6^

Research in social psychology suggests that many people believe that they (and others) have a true self that is constituted by a deep ‘essence’, that they need to discover in order to live authentically (Christy et al. 2017). On this essentialist approach, the true self is understood to be constituted by discrete, biologically based, immutable, informative, and consistent characteristics (Haslam et al. 2004; Newman et al. 2014; Strohminger et al. 2017). To determine whether a change to a psychological feature is authentic on this approach, one must consider the *content* of the change; is the changed psychological feature congruous with the underlying true self? Accordingly, there are direct parallels between this understanding of authenticity and Witt et al.’s core-periphery model. This essentialist understanding of the true self conceives of the self in a broadly static sense, in contrast to the dynamic conceptions of narrative and relational identity outlined in the previous section. For this reason, it has been subject to considerable criticism by those who doubt the possibility of such an extant static self (Baylis 2013; DeGrazia 2005, pp. 233–234; Newman et al. 2014).

This is perhaps somewhat surprising, given the congruity of this approach with highly influential trait theories in personality psychology. Moreover, even if essentialism can be subject to damning theoretical criticisms, there is evidence to suggest that many people, including patients, employ such a view of themselves. Indeed, as Alexandre Erler and Tony Hope have highlighted in a recent discussion, those with mental disorders often appeal to essentialist conceptions of authenticity to help guide their choices and commitments (Erler and Hope 2015, p. 230).

However, it is also possible to understand authenticity in a more dynamic sense, as a product of one’s own creation (DeGrazia 2005, Chapter 3). On this existentialist approach, it is suggested that the self undergoes considerable change in projects of self-creation, but that this is in fact constitutive of the authentic life, if the agent herself actively identifies with those changes in some way. To determine the authenticity of a change to a psychological feature on this approach, one must focus on the *process* by which the change came about (rather than its content); for instance, it might be claimed that changes to one’s character can only be authentic it the result of an autonomous decision to bring about such change (DeGrazia 2005). Whilst the answer to the causal question in the context of DBS might again be important on such process-oriented approaches, it need not be; changes to psychological features that are developed and maintained unreflectively or sub-consciously in response to one’s treatment trajectory may be just as concerning as those that are (supposedly) directly induced by DBS.

Finally, it is also possible to think about the true self and authenticity in accordance with a dual-basis framework that incorporates elements of both of these apporaches (Pugh et al. 2017). This framework incorporates the essentialist notion that certain elements of an individual’s character are more or less fixed, but also the existentialist notion that individuals can choose which of these more or less fixed elements to bring to the fore, and which to downplay in projects of self-creation. On this approach, both the process of the change (emphasised by Schechtman and DeGrazia) and the content of the change (emphasised by Witt and colleagues’s core-periphery model) matter. Yet, even quite radical changes of the sort that Witt and colleagues describe as ‘paradigm shifts’ need not threaten authenticity on this understanding (Witt et al. 2013). However, such changes cannot be entirely wholesale; projects of self-creation must be grounded by relatively stable diachronic values and beliefs that render these projects intelligible (Pugh et al. 2017).

These different approaches offer different answers to the question of how to distinguish changes that matter from those that do not.^7^ Crucially, the claim that “a patient has become a different person” or that they have “changed personality” does not entail that the change is inauthentic on *any* of these theories. This is most obviously true in the case of the existentialist and dual-basis views. However, it is also true on the essentialist approach in cases where the person was not previously living in accordance with their true self; in such cases, significant change may have allowed the patient to become their true, essentialist self, or to perhaps rediscover it after it has been overshadowed by years of chronic illness.

It might be claimed that this paper has thus far been considering authenticity in an overly atomistic sense, as something that is established solely by the individual herself. It is clearly true that any adequate theory of authenticity should also allow for the undeniable relational influences on an individual’s conception of their authentic self, and it is possible to incorporate this insight into the different understandings of authenticity outlined above, by incorporating considerations of how others perceive the individual (Baylis 2013), and the kinds of group identities that might be important to them (Bluhm et al. 2019).

However, relational theorists might deny that this strategy can sufficiently attend to the fundamental way in which relationality is built into these concepts. One relational strand in the literature on DBS denies that the true self is something that is either discovered or created (Goddard 2017; Mackenzie and Walker 2014). Instead, on this approach the true self and authenticity are best conceived as “emergent phenomena” that result from the exercise of competencies associated with autonomous agency, which are developed and exercised in the context of social relationships (Goddard 2017). On this approach, the claim that DBS changes identity directly is a red herring. Considerations of autonomy are theoretically prior to those of identity, insofar as the latter emerges from the exercise of the former, and any change to identity must thus be considered through the “lens of agency” (Goddard 2017).

This relational approach has significant implications for the ethical questions associated with autonomy in the context of DBS. Accordingly, this paper shall delay further analysis of this particular theory until the final section in which it considers the concepts of autonomy and agency in greater detail. Here though, it is possible to schematise the different approaches to the concepts surveyed in this section as follows. Whilst there is a considerable degree of overlap between all of these concepts, the table is colour coded to indicate those that share particular affinities (Table 1).

**Table 1:**
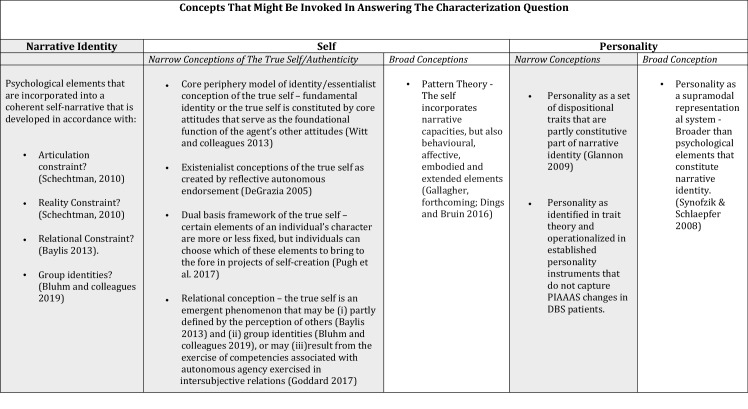
Further concepts that might be invoked in answering the characterization question

To conclude this brief sketch of different broad approaches to understanding the concepts of personality, authenticity and the self, this paper shall elucidate some of the implications of these concepts for well-being and the principle of beneficence, and some considerations about the role different concepts ought to play in the ethical debate pertaining to DBS.

An obvious starting point for elucidating the normative implications of changes to personality, self, authenticity and/or narrative identity is to focus on the subject’s experiential well-being. On this approach, it might seem natural to assume that changes to personality self, authenticity, or narrative identity will be detrimental to experiential well-being, and that changes which are ‘liked’ will not be damaging to the patient’s sense of continuity of self. Indeed, as suggested above, those who adopt broad theories of the self and personality may suggest that this is the most significant normative question in this context. Yet such a straightforward reading would be misleading. It is certainly true that some patients do report distress at self-observed changes following DBS treatment. However, data from Gilbert and colleagues and De Haan and colleagues suggests that patients do not always experience these change as negative. More importantly though, their data supports the idea that merely considering whether a change is liked by the patient is not sufficient for fully elucidating the moral significance of changes following DBS (de Haan et al. 2017).

In Gilbert et al.’s study investigating patient attitudes to DBS treatment for Parkinson’s disease, Gilbert and colleagues noted that patients can experience self-estrangement following treatment, but that this phenomenon can involve either “deteriorative estrangement” or “restorative estrangement” (Gilbert et al. 2017). Both forms of estrangement involve experiencing an involuntary shift in one’s character, in this case following DBS treatment. In deteriorative estrangement, this is experienced as a deterioration of the patient’s self, and is qualitatively experienced as a sense of powerlessness; in contrast, in restorative estrangement, the shift in one’s character is experienced as restoring elements that were central to the self that existed prior to the onset of disease (Gilbert et al. 2017). They suggest that there is a correlation between how patients preoperatively perceive themselves with respect to their illness, and their experiences of estrangement (Gilbert et al. 2017).

This data does not just show that self-estrangement can be experienced as positive or negative, as liked or not liked. Rather, the difference between these two types of estrangement indicates that patients’ own self-conceptions are playing a crucial role in their experience of treatment. This suggests that simply asking patients whether they like the changes that they have undergone will only furnish one with a superficial understanding of why patients believe that these changes are morally significant.

Accordingly, feelings of estrangement are not necessarily negative. Moreover, a feeling of authenticity does not necessarily track positive subjective well-being (Kraemer 2013). Support for this point can be found in De Haan’s study, which suggests that even changes that are perceived as negative by the patient may nonetheless be judged to reflect ‘who the individual is’ (de Haan et al. 2017). For instance, this might occur because the patient identifies their true self as the self in the absence of disease, but nonetheless struggle with elements of living as a healthy individual. In terms of experiential well-being then, changes to self, personality, narrative identity, and authenticity can be ambivalent.

The data from these studies suggest that traditional bioethical concepts of beneficence and non-maleficence are unlikely to apply straightforwardly in cases where an individual has undergone a change to PIAAAS following DBS. The reason for this is that there is considerable room for disagreement about the role that authenticity and self-estrangement play in well-being. That said, on a suitably developed theory of the relationship between authenticity and well-being, it might be possible to weigh the prudential costs (or benefits) of experiences of estrangement against the other prudential costs and benefits of treatment. In this respect, a decision to undergo DBS would be similar to decisions to undergo any medical procedure that posed a risk of severe side-effects. There would of course still be difficult questions to answer in such a weighting. Nonetheless, it is widely accepted that competent patients have the right to make their own autonomous decisions about these matters when choosing to provide consent to medial treatments.

However, the above empirical data arguably hints towards deeper normative considerations of the sort that broad conceptions of self and personality may not be well-placed to capture. The final section of this paper shall attempt to further elucidate deeper normative implications that these experiences may have with regards to two further concepts incorporated into Gilbert and colleagues’s category of PIAAAS: agency and autonomy.

## Agency and Autonomy

Agency is commonly construed in a narrow sense to pertain to an individual’s exercise of their ability to *act* in the world. Even this basic understanding is sufficient to elucidate the fact that DBS can clearly influence agency; indeed, this may be its therapeutic aim. For instance, in the context of movement disorders, the therapeutic intention of DBS is to alleviate motor impairments that greatly reduce an individual’s ability to act. Similarly, in the context of OCD, DBS may enable agency by alleviating debilitating compulsions that would otherwise impede the patient from acting in certain ways (de Haan et al. 2017).

Whilst agency understood in this sense is concerned only with action, it would be a mistake to entirely distinguish it from considerations of the self, personality, authenticity and (narrative) identity. The reason for this is that an individual’s beliefs about their self-efficacy as an agent can impact on a range of psychological features, particularly on intention (Bagozzi 1992); accordingly, significant changes to an individual’s abilities may plausibly have important implications for how they understand themselves (Bandura et al. 2001). Indeed, de Haan and colleagues hypothesise that some patients in their study believed that DBS had changed them as a person *because* of the way in which it enhanced their agency (de Haan et al. 2017).

However, in the ethical debate concerning DBS, agency has also been construed in a far broader sense. For instance Baylis claims that DBS primarily poses a threat to agency rather than identity, defining agency as:

… the ability to make informed and rational choices—as when a person’s actions do not flow from her intentions or beliefs but rather are the result of direct brain manipulation. (Baylis 2013, p. 524)

This approach seems to lump agency together with concept of autonomy. For instance, Baylis’ understanding of the agency that DBS threatens is strikingly similar to a widely endorsed definition of procedural autonomy employed in bioethics, according to which a person is autonomous with respect to a particular understanding if they perform it (1) with substantial understanding; (2) intentionally; (3) in the absence of controlling influence, including manipulation (Beauchamp and Childress 2009). Individuals are only able to make autonomous decisions if they have the capacities required to meet these conditions.

The previous section explored the moral significance of the concepts of authenticity, narrative identity and self with respect to their implications for individual well-being, and the bioethical principles of beneficence and non-maleficence. However, the moral significance of autonomy in bioethics is typically understood to be distinct from considerations of well-being. This is reflected by the distinct principles of autonomy and beneficence in the influential four principles approach, and also by the thought that patients should typically have the right to refuse treatments that are in their best interests. In respecting an agent’s autonomy, one does not only view that person as a subject of well-being, but as a *moral* subject whose preferences should be understood to have considerable (though not complete) authority with respect to those matters that centrally concern her. However, this is not to say that considerations of autonomy and well-being are entirely distinct (Pugh 2020). Indeed, it might be claimed in a broadly Millian tradition that autonomy is partly constituitive of well-being, according to which an individual’s own mode of laying out his existence is prudentially best “… not because it is the best, but because it is his own mode” (Mill 2003, p. 131).

To what extent should we say that DBS can pose a threat to autonomy, and is our current understanding of the deeply entrenched principle of respect for autonomy fit to capture this threat? Answering this question requires a great deal of nuance, particularly when we consider more extreme cases of changes to PIAAAS. First, it is important to observe that autonomy can be understood in both a local and a global sense. In the latter sense, autonomy is conceived as a property that individuals instantiate over the course of long periods of time, including their lives as a whole. However, autonomy can also be conceived in a local sense, as a property of individuals at a particular point in time, with respect to a specific decision or action. Requirements of informed consent, which largely enshrine the particular normative significance attributed to autonomy in bioethics, are primarily concerned with autonomy in this local sense. Further, whilst the capacities that constitute autonomy may admit of degrees, medical law implicitly treats it a range property; a particular local decision will only qualify as autonomous in the manner that betokens valid consent if the individual exercises the competencies relevant to autonomy to a certain threshold.

Crucially, there are some circumstances in which the side-effects of DBS treatment can diminish the recipient’s capacity to provide valid informed consent. For instance, this would be the case if treatment reduced the patient’s ability to retain and understand relevant information, and to make a decision on its basis (Klaming and Haselager 2010). A widely discussed case study described by Leentjens et al. (2004) provides a particularly vivid example. In this case, a patient developed a severe manic state that impaired his decision-making capacity whilst undergoing stimulation that nonetheless ameliorated his severe motor impairment. Whilst off stimulation, the patient chose to continue with long-term treatment, even though this meant that he would need to be committed to a psychiatric ward.

In such cases, DBS may affect what might be described as the cognitive elements of the patient’s decision-making capacity, in so far as it diminishes their capacity to understand, retain, and use material information (Pugh 2020). However, DBS might also have a pernicious effect on other important elements of autonomy. On some approaches, if an individual’s decision-making is to be truly autonomous, it must be appropriately connected to their evaluative judgments (Buchanan and Brock 1989, p. 29), and in particular their authentic values (Pugh 2020). On this somewhat broader conception of autonomy, autonomy requires a greater range of capacities than those highlighted by the standard account; it requires the emotional and critical capacities associated with being a valuer.

I have already explained how agency understood in a narrow sense may be related to the concepts of self, authenticity, and personality. Furthermore, on accounts of agency construed as autonomy in the broader sense, there may be a further close relationship between autonomy and at least certain accounts of authenticity and narrative identity. The reason for this is that some accounts of the latter concepts particularly emphasize the importance of evaluative identification with either elements of the self, or of one’s narrative.^8^ For instance, David DeGrazia draws a close relationship between the authenticity and autonomy in this way, by proposing an autonomy-based understanding of authenticity, where authentic self-creation amounts to the “autonomous writing of one’s self-narrative” (DeGrazia 2005, p. 112), where autonomy requires identification of the sort that ensures that “…autonomous action will flow from one’s values” (DeGrazia 2005, p. 103).

There may be a concern that linking these concepts in this way risks conflating considerations of authenticity and autonomy. Indeed, some relational theorists of autonomy have objected to these accounts partly on this basis (Goddard 2017). This critique shall be considered in greater detail at the end of this section. However, it should again be acknowledged that one can incorporate insights from a relational approach to autonomy in the context of DBS, recognising that autonomy is a “characteristic of agents who are emotional, embodied, desiring, creative, and feeling, as well as rational, creatures”, without abandoning the claim that self-narratives and evaluative judgments can play a significant role in a theory of personal autonomy (Gallagher forthcoming).

If one accepts the claim that locally autonomous decisions should be grounded by the agent’s own values and judgments, then one straightforward way in which DBS can threaten autonomy is if it leads the recipient to become motivated to perform behaviors that are contrary to their values. For instance, in a case described by Paresh Doshi and Pranshu Bhargava, an elderly patient undergoing DBS of the subthalamic nucleus developed aggressive hypersexuality as a side effect. Once his urges were, satisfied, the patient returned “back to his normal self,” and admitted that he could not control his unwanted urges (Doshi and Bhargava 2008). Such cases, and those in which patient’s developing other uncontrolled behaviours such as pathological gambling are plausible examples of patients whose autonomy has been affected by undergoing DBS treatment. Indeed, they are broadly comparable to Harry Frankfurt’s example of the ‘unwilling addict’, who is presented as a paradigm case of an individual who lacks autonomy (Frankfurt 1971). Equally though, the intended therapeutic effect of DBS in psychiatric applications may be to enhance an agent’s ability to act in accordance with their values, to overcome pathologically impulsive behaviours, and to exert top-down control in their local decision-making (Maslen et al. 2015b).

In these cases, individuals develop new behavioural traits that they do not endorse. However, arguably more complex cases arise when DBS may plausibly have an effect on the underlying values that inform an individual’s endorsements, and their decision-making more broadly. For instance, apathy has been observed as a postoperative symptom of STN stimulation, (Voon et al. 2006) and apathy may plausibly be understood to threatens an agent’s ability to be moved to express or act on their values. Furthermore, in psychiatric applications in which DBS is intended to alter dysfunctional emotional processing or reward processing, it seems plausible to hypothesise that DBS may affect the patient’s evaluative stance; indeed, this may be the professed aim of the treatment (de Haan et al. 2017; Pugh et al. 2017).

Accordingly, there is a good case for distinguishing between the threat that DBS may pose to autonomy, by virtue of (1) the effect that it may have on the individual’s behavioral traits, and (2) the effect that it may have on the patient’s evaluative stance (Pugh et al. 2017). The former is morally significant, because it leads an agent to perform non-autonomous actions that may also be detrimental to their global autonomy, their own well-being, and perhaps the well-being of others; however, it does not threaten the patient’s status as a subject capable of making their own locally autonomous treatment decisions about whether the benefits of DBS treatment outweigh the risks of such side-effects. In contrast, if stimulation leads to the development of inauthentic values, or impairs other autonomy competencies outlined by relational theorists, then this is arguably of greater normative significance, in so far as it shifts the very grounds of the patient’s capacity to make locally autonomous treatment decisions.

The largely reversible nature of the stimulation-dependent elements of DBS treatment makes this particularly significant. DBS is an-on-going diachronic treatment process that requires the assessment of capacity, and the solicitation of consent across a number of different points in the recovery trajectory (Pugh 2019). Changes to a patient’s underlying values across time could thus potentially have considerable implications for how we should operationalize the principle of respect for autonomy in this context. More specifically, it is not clear that current understandings of the concept of autonomy in medical ethics will always provide sufficient guidance about whether doctors ought to respect treatment decisions that the patient makes on and off stimulation, if the two conflict (Maslen et al. 2015a).

Fortunately, the limited empirical literature on patient attitude towards DBS for OCD suggests that although patients change their behavioural traits following treatment, they do not appear to fundamentally change their values in this way (de Haan et al. 2017). Indeed, the above distinction between traits and values is echoed in the existing empirical data. For example, as highlighted above, de Haan and colleagues suggest that some patients may believe that the facilitation of agency (in the narrow sense) that DBS elicits changes them as a person. However, they also note that other patients instead believe that one only changes as a person if one fundamentally changes one outlook on life, and that DBS has not changed them in this way (de Haan et al. 2017, p. 18).

Even if DBS did lead to a change in one’s values, this need not undermine autonomy on the theories that emphasize the importance of evaluative judgments to autonomy. Here there is a considerable overlap with the literature on authenticity; as discussed above, changes to one’s evaluative stance following DBS need not be inauthentic. However, in order to assess these changes, it is crucial to have an account of when changes to a person’s approach to valuing are compatible with retaining their capacity as an autonomous decision-maker. As explained above, approaches to the concept of authenticity might plausibly offer answers to this question. One theme that emerges from this debate is the different degrees of emphasis that these theories place on the first-person perspective in ascertaining authenticity. The theoretical disagreement here has stark practical implications in the context of DBS, as the recipient’s own perspective of their authenticity can be in stark contrast to the assessment of their loved ones.

For instance, in his existentialist approach to authenticity, DeGrazia explicitly privileges the subjective perspective. He writes: “With regards to the question ‘who am I’—Only an answer that favours the first person standpoint does justice to such a first-person question” (DeGrazia 2005, p. 84) and that he finds “… a privileging of the first person perspective the only reasonable option” (DeGrazia 2005, pp. 84–87). Nonetheless, relational theorists argue that this approach is ill-equipped to deal with cases involving the sort of conflict outlined here, in comparison to the relational understanding, according to which there is no narrative other than that for which one can get uptake (Baylis 2013; Goddard 2017, p. 330).

It is undeniable that human beings are relationally situated creatures whose decisions are subject to the influence of others. This is a key insight of the relational approach that any plausible approach to understanding the different elements of PIAAAS must be able to accommodate. But there is still a legitimate question about the extent to which these influences should be weighed against the first person perspective that DeGrazia privileges. The approach that one adopts to this question has important ramifications with regards to the potential effects of DBS on locally autonomous decision-making. The reason for this is that if it is true that third parties and group identities have considerable scope in establishing the agent’s true values, then this may shift the concept of autonomy under discussion towards a substantive rather than procedural conception (Dive and Newson 2018; Mackenzie and Walker 2014). On such substantive conceptions, the autonomy of a patient’s decisions is to be determined by its content (in this case, whether the content is compatible with third party views on what is *valuable*), rather than the procedure by which they came to make the decision (on the basis of what they *value*).

The suggestion that granting significant authority to third-party assessments of these features risks a slide into a substantive conception of autonomy is made all the more plausible by research suggesting that beliefs about both one’s own true self, and the selves of others are perceived positively; the true self is typically identified as good and moral by whoever is assessing it (Newman et al. 2014). If this is so, then disagreements about whether a change to a person’s values (or their evaluative competencies) is compatible with autonomy on the relational approach may simply boil down to fundamental disagreements about the good; the decision will be autonomous/authentic if it is congruous with third party assessments of the good.

Whilst this debate between different approaches to autonomy cannot be settled here, it is prudent to acknowledge the challenge that this theoretical question raises in the context of DBS. Whilst relational theories clearly capture important insights, the possibility that individuals may disagree with third parties about the nature of the good plausibly helps to ground much of what appears to be normatively significant about autonomy. One plausible concern that might be raised in this context is that substantive accounts threaten the abandonment of the Millian thought that there is a distinct value in carrying out one’s own mode of existence, and performing experiments in living, raising instead the spectre of paternalistic interference (Pugh et al. 2017). Accordingly, the challenge is how to balance the ways in which third parties can help us interpret the extent of authentic evaluative change following DBS (Nyholm 2018), whilst also maintaining the individual’s ability to value their own judgment, and form the sort of normative commitment to themselves that relational theories of autonomy highlight (Goddard 2017).

## Conclusion

This paper has highlighted some of the considerable disparities in the understanding of central philosophical concepts that have been invoked in the neuroethical debate concerning DBS and alterations to PIAAAS. It has outlined various ways in which these concepts might be defined, how they might be related on some conceptions, and the normative implications of these different approaches.

It is possible to draw some important practical conclusions from this analysis regarding how the field might move forward in its attempt to bridge the gap between the empirical and normative discussions in this area. First, whilst empirical investigations into the effects of DBS may need to lump together the different concepts discussed here in order to fully capture the nature of patients’ experience, there is little justification for theoretical neuroethical analyses of these phenomena following the same strategy. Ethicsts should strive for greater clarity with respect to not only the particular concepts that they are invoking in their analyses, but also the scope of their normative significance. This is not to say that ethicists should agree about the nature and value of these concepts; however, they should reach a greater degree of shared understanding about the particular nature of what it is they are disagreeing about.

Yet neuroethics has a lot to offer the further empirical investigation of changes to PIAAAS following DBS treatment. First, investigators should adopt a broad church in investigating this phenomenon. Many of these concepts can be understood in broad terms, incorporating various elements of the patient’s lives, including third party assessments of the patient’s beliefs and values. Accordingly, in order to fully understand the nature of these changes and their normative significance, the empirical investigation of them must be far reaching, both in terms of the points of investigation, and the depth of the investigation itself. With respect to the former, understanding of this phenomenon would be supplemented by interview studies involving patients, carers, and families at different points across the treatment trajectory (including prior to the initiation of treatment). With respect to the latter, studies should aim to go beyond the patient’s reports of new behaviours, and even the patient’s feeling about these changes, to a deeper consideration of the nexus of the patients wider beliefs and values that inform their own judgments on this matter, the strategies with which they cope with the changes they experience, as well their own understanding of what they think it is to become a different person, and why that might matter.

Finally, it is also crucial that empirical investigations into this phenomenon are themselves value neutral. Early investigations of the topic were guilty of assuming that changes following treatment were instances of social maladjustment (Kraemer 2013). Such an approach lacks philosophical support, and has now been invalidated by data showing the different ways in which patients evaluate and experience change. To progress further, empirical analysis should aim to broaden the scope of its investigation beyond the question of whether changes matter solely for the patient’s experiential well-being, to the broader range of normative implications that this paper has highlighted. Accordingly, whilst broad accounts of self and personality that allow for a comprehensive picture of the potential effects of DBS treatment are undoubtedly of interest, there are also good reasons to employ accounts with a narrower focus that emphasize changes of particular normative significance.

## Footnotes

1 For criticisms of Gilbert and colleagues arguments, see Bluhm and Cabrera (2018), Erler (2019), Pugh et al. (2018) and Snoek et al. (2019).

2 For a small sample, see Baylis (2013), Goddard (2017), Lipsman and Glannon (2013) and Schechtman (2010).

3 The reason why psychological continuity is not sufficient for identity is that an individual person might plausibly be psychologically continuous with two distinct other persons in thought experiments involving fission. This complexity need not concern us here.

4 Parfit famously argues that numerical identity is not what matters in survival. However, these complications concerning the possibility of fission cases (see previous note) need not concern us here.

5 Lipsman and Glannon (2013, p. 466) suggest that DBS for schizophrenia could theoretically threaten numerical identity if it induced psychotic states.

6 For a discussion of whether the true self should be construed in a synchronic sense in the context of DBS, see Nyholm and O’Neill (2016), Pugh et al. (2017).

7 Unlike Goddard’s relational approach outline below, it also allows for the thought that changes unrelated to the exercise of agency per se may have normative significance. One candidate for such a change may be alterations to an individual’s hedonic likings and dislikings following treatment. For one example of this, see Mantione’s report of a patient developing different musical tastes under stimulation (Mantione et al. 2014).

8 There may be other ways of relating the two concepts. For instance, Kraemer suggests that a claim by one of the patient interviewed by Schupbach suggest that the patient understood authenticity to correlate with autonomy, construed in terms of agential independence (Kraemer 2013, p. 491).
